# Regulation of Hippo, TGFβ/SMAD, Wnt/*β*-Catenin, JAK/STAT, and NOTCH by Long Non-Coding RNAs in Pancreatic Cancer

**DOI:** 10.3389/fonc.2021.657965

**Published:** 2021-06-09

**Authors:** Ammad Ahmad Farooqi, Sawera Nayyab, Chiara Martinelli, Rossana Berardi, Hector Katifelis, Maria Gazouli, William C. Cho

**Affiliations:** ^1^ Institute of Biomedical and Genetic Engineering (IBGE), Islamabad, Pakistan; ^2^ Department of Biotechnology, Faculty of Science, University of Sialkot, Sialkot, Pakistan; ^3^ Independent researcher, Como, Italy; ^4^ Università Politecnica delle Marche-Ospedali Riuniti Ancona, Ancona, Italy; ^5^ Laboratory of Biology, Medical School, National and Kapodistrian University of Athens, Athens, Greece; ^6^ Department of Clinical Oncology, Queen Elizabeth Hospital, Hong Kong SAR, China

**Keywords:** lncRNA, apoptosis, signaling pathways, microRNA, pancreatic cancer

## Abstract

Rapidly evolving and ever-increasing knowledge of the molecular pathophysiology of pancreatic cancer has leveraged our understanding altogether to a next level. Compared to the exciting ground-breaking discoveries related to underlying mechanisms of pancreatic cancer onset and progression, however, there had been relatively few advances in the therapeutic options available for the treatment. Since the discovery of the DNA structure as a helix which replicates semi-conservatively to pass the genetic material to the progeny, there has been conceptual refinement and continuous addition of missing pieces to complete the landscape of central dogma. Starting from transcription to translation, modern era has witnessed non-coding RNA discovery and central role of these versatile regulators in onset and progression of pancreatic cancer. Long non-coding RNAs (lncRNAs) have been shown to act as competitive endogenous RNAs through sequestration and competitive binding to myriad of microRNAs in different cancers. In this article, we set spotlight on emerging evidence of regulation of different signaling pathways (Hippo, TGFβ/SMAD, Wnt/*β*-Catenin, JAK/STAT and NOTCH) by lncRNAs. Conceptual refinements have enabled us to understand how lncRNAs play central role in post-translational modifications of various proteins and how lncRNAs work with epigenetic-associated machinery to transcriptionally regulate gene network in pancreatic cancer.

## Introduction

It has been reported that only 1–2% of RNAs encode for proteins and that the great majority of them falls into the non-coding category, comprehending a large number of different structural (ribosomal RNAs, rRNAs and transfer RNAs, tRNAs) and regulatory RNAs (small conditional RNAs, scRNAs; microRNAs, miRNAs; small nucleolar RNAs, snoRNAs; long non-coding RNAs, lncRNAs) (1). Regulatory RNAs can be divided into small, medium, and long non-coding RNAs (2). LncRNAs sequences are poorly conserved and thus their genomic identification results often difficult (3). They are defined as long RNA transcripts (> 200 nucleotides) not translated in proteins (4), which are involved in the regulation of transcriptional processes by modulation of other non-coding RNAs (ncRNAs) (5). These nucleic acids are responsible also for regulating gene expression at transcriptional and post-transcriptional levels (6, 7). Even though largely debated, recent studies performed exploiting ribosome sequencing (Ribo-seq) and mass spectrometry have revealed their possible translation into proteins (8). Many researches have focused on unraveling the functions of ncRNAs and a careful classification based on their characterization has been established. In recent years, thanks to RNA sequencing and innovative methods, a great number of categories have been identified (9).

LncRNAs can originate from their own/shared promoters, from enhancers and intergenic regions and in specific cell-types upon stimuli. For example, human pancreatic *β*-cells contain thousands of lncRNAs that can be controlled during differentiation, maturation and upon glucose dynamic changes and are responsible for regulating gene expression in diabetes. Although their function is still under investigation, many techniques have been used for unraveling lncRNAs intracellular localization, structure and functions (10–12).

Different mechanisms that are involved in their biogenesis and peculiar subnuclear structures, called paraspeckles, have been identified. They localize in proximity of nuclear enriched abundant transcript 1 (NEAT1) lncRNAs, specific transcripts lacking introns. Recent studies identified four paraspeckle proteins required for their formation during NEAT1 synthesis and processing. Importantly, paraspeckles have been also involved in modulation of gene expression mediated by lncRNAs (13).

Interestingly, lncRNAs can be transcribed by many regions in the genome, including promoters, enhancers or long primary transcripts. Processing of lncRNAs can be carried out by ribonuclease P for generating mature 3′ ends capped by small nucleolar RNA-proteins or by formation of circular structures (14). LncRNAs can be transcribed as promoter upstream transcripts (PROMPTs) (15), enhancer RNAs (eRNAs) (16), long intervening/intergenic ncRNAs (lincRNAs) (17). When they are transcribed by RNA polymerase II (Poly II), they are produced as medium length lncRNAs with short half-life and targeted by a special nuclear degradation complex called RNA exosome. This process put their gene regulatory activity at high risk.

The most studied category of lncRNAs is lincRNAs, transcribed by Pol II from intergenic regions. These transcripts contain multiple exons and similarity to mRNAs (18), even though they present very different features: they do not possess encoding sequences, they present tissue specific expression, localize at the nucleus and have specific functions (19, 20). Other lncRNAs transcribed from the natural antisense transcripts called NATs (21).

Recently, it has been shown that lincRNAs present few histone marks and transcription factors attached to their promoters and are less spliced respect to mRNAs (22). Interestingly, some lncRNAs can be processed from long transcripts to obtain structures without 5′cap or 3′ adenosine tails (23).

LncRNAs are involved in the transcription modulation mediated by control of gene expression by attaching to DNA binding proteins and Pol II. They can interfere with many cellular mechanisms, such as cell proliferation, differentiation and development (5). Interestingly, they have been demonstrated to be associated to tumorigenesis, when mutated or dysregulated. They are involved in the onset of many types of tumors, such as colorectal, lung, liver, pancreatic, and ovarian cancer (24, 25). Some studies have shown their involvement in several diseases (26).

To provide a comprehensive overview of the interplay between lncRNAs and signaling pathways in pancreatic cancer, we have partitioned this article into three sections. In the first section, we focus on the elucidation of the molecular mechanisms used by lncRNAs to modulate signaling pathways in pancreatic cancer. This section exclusively deals with regulation of Hippo, TGF*β*/SMAD, Wnt/*β*-catenin, Notch, JAK/STAT and TRAIL-driven pathways by lncRNAs in pancreatic cancer. In the second section, we provided a list of individual lncRNAs reportedly involved in the regulation of protein networks in pancreatic cancer. In the last section, we outlined the list of lncRNAs which served as sponges for miRNAs and sequestered target mRNAs away from inhibition by miRNAs.

## Mechanism Based Regulation of Pancreatic Cancer by LncRNAs

LncRNAs mediated regulation of pancreatic cancer is highly intricate. Different proteins have been shown to regulate expression of lncRNAs. Likewise, lncRNAs have been reported to work with methylation specific machinery to activate or repress myriad of genes. Moreover, lncRNAs have also been noted to regulate post-translational modifications of different proteins. In this section, we exclusively focus on regulation of Hippo, TGF*β*/SMAD, Wnt/*β*-catenin, Notch, JAK/STAT and TRAIL-driven pathways by lncRNAs in pancreatic cancer.

### Regulation of Hippo Pathway by Long Non-Coding RNAs

**Figure 1:**
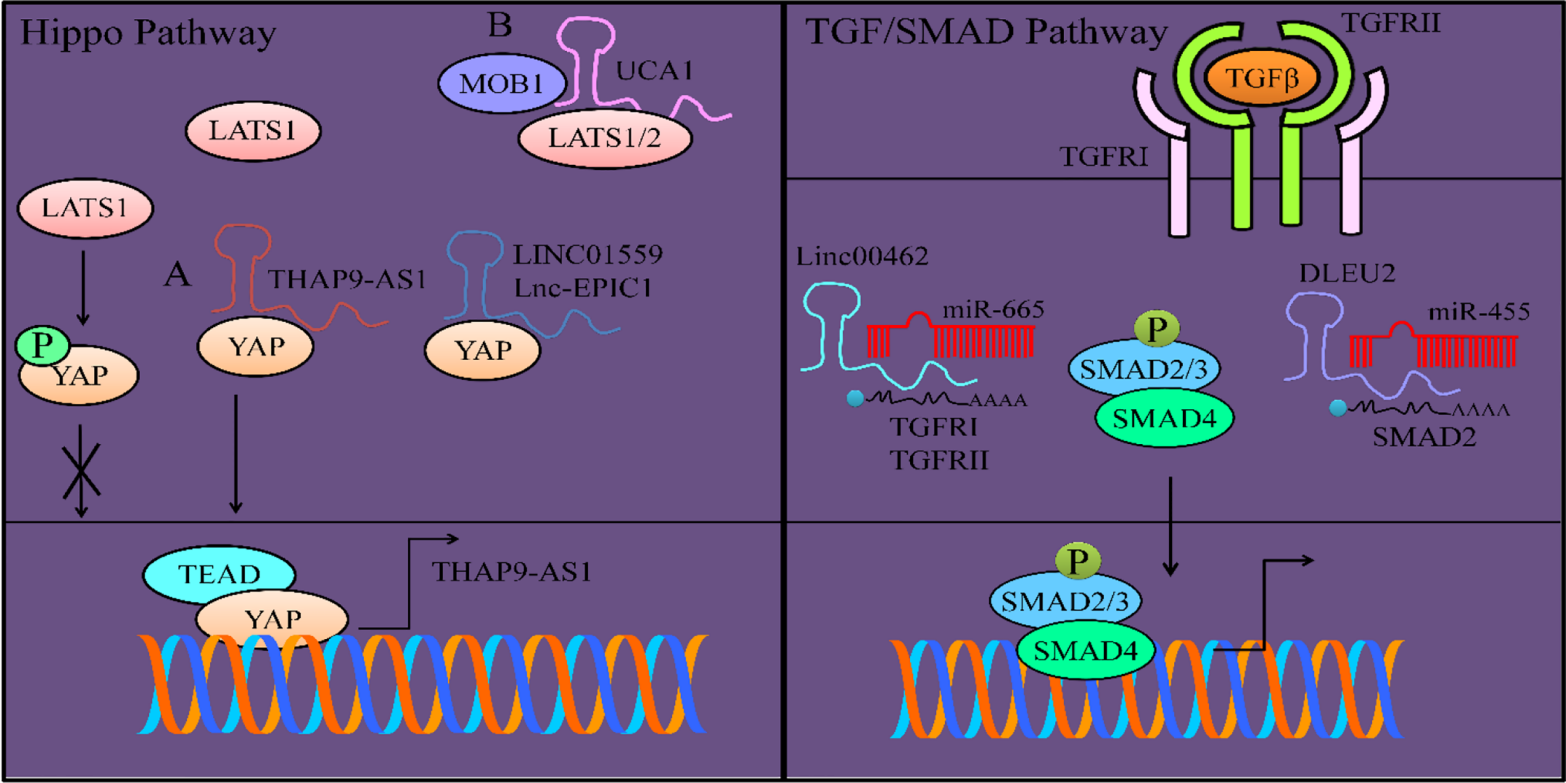
Regulation of Hippo pathway. LATS1 mediated phosphorylation of YAP prevented its nuclear accumulation. However, **(A)** THAP9-AS1 inhibited LATS1 mediated phosphorylation of YAP and promoted its nuclear accumulation. YAP interacted with TEAD and transcriptionally upregulated THAP9-AS1. LINC01559 and UCA1 also inhibit phosphorylation of YAP and promote its nuclear accumulation. **(B)** UCA1 formed a complex with MOB1 and LATS1/2 and not only inhibited MOB1-mediated activation of LATS1/2 but also blocked phosphorylation of YAP. TGFβ/SMAD pathway is regulated by lncRNAs. Linc00462 enhanced the expression of TGFRI and TGFRII by interfering with miR-665-mediated targeting activity. DLEU2 also served as an oncogenic lncRNA and inhibited miR-455-mediated targeting of SMAD2.

When the Hippo pathway is activated, LATS1/2 kinases (Large tumor suppressor-1/2) phosphorylate and inactivate YAP (Yes-associated protein) and transcriptional coactivator having PDZ-binding motifs (TAZ), the two characteristically unique downstream transducers that mediate transcriptional output of the Hippo-driven transduction cascade. Phosphorylation of YAP at 127^th^ serine residue by LATS1 is a classical post-translational modification which induces cytoplasmic retention of YAP to inhibit its activity (27). THAP9-AS1 knockdown resulted in an increase in the phosphorylation of YAP at 127^th^ serine residue. THAP9-AS1 increased expression of YAP by sponging miRNA-484 away from YAP. Series of experiments revealed that WW1/2 domain of YAP was essential for direct interaction with LATS1 to induce phosphorylation and subsequent retention of YAP within cytoplasm (27) (**Figure 1**). THAP9-AS1 interacted with YAP and blocked LATS1-YAP association. Co-immunoprecipitation assays revealed that knockdown of THAP9-AS1 potentiated the interactions between LATS1 and YAP. Although YAP/TAZ are transcriptional co-activators, they do not have DNA-binding domains. Therefore, YAP/TAZ need binding partners and usually bind with transcriptional factors such as TEAD1-4 for modulation of expression of target genes. YAP/TEAD1 complex transcriptionally upregulated the expression of THAP9-AS1 (**Figure 1**) (27).

It is exciting to note that most of the lncRNAs (LINC01559 and UCA1) inhibit phosphorylation of YAP and promote its nuclear accumulation.

LINC01559 interacted with YAP, inhibited YAP phosphorylation, and enhanced YAP/induced transcriptional activities in pancreatic cancer cells (28).

YAP1 maintained the expression of MYC, whereas knockout of YAP1 caused considerable downregulation of MYC that resulted in growth arrest of pancreatic cancer cells and apoptosis (29). Lnc-EPIC1 interacted with YAP1 in pancreatic cancer cells. Lnc-EPIC1 lost its ability to promote proliferation and growth of YAP1-silenced pancreatic cancer cells (29).

Likewise, UCA1 significantly enhanced the invasive ability of PANC-1 cells (30). LATS1/2 requires a co-activator protein to achieve full activation. MOB1 (Mps One binder 1) is essential for the activation of LATS1/2. UCA1, formed a complex with LATS1, MOB1 and YAP (**Figure 1**). UCA1 promoted nuclear translocation of YAP in PANC-1 cells. Additionally, YAP stimulated the expression of UCA1 in pancreatic cancer cells (30). Overall, UCA1 and YAP promoted invasion of pancreatic cancer cells.

These interesting findings provided substantial evidence that different lncRNAs inhibited YAP phosphorylation and potentiated YAP-driven signaling to promote pancreatic cancer.

MST1 and MST2 promoted the phosphorylation of LATS1 and LATS2 (31). MST1 was found to be directly targeted by miR-181c-5p. miR-181c-5p promoted chemoresistance of pancreatic cancer cells through inactivation of the Hippo signaling. GAS5 interfered with miR-181c-5p-mediated inhibition of MST1 in pancreatic cancer cells. Tumor growth was significantly reduced in mice inoculated with GAS5-overexpressing PANC-1 cells (31).

### Regulation of TGF*β*/SMAD Signaling

TGF*β*/SMAD signaling has been shown to play significant role in the onset and progression of pancreatic cancer. Here we discuss how different lncRNAs regulate TGF*β*/SMAD to promote pancreatic cancer.

Linc00462 considerably enhanced invasive potential of pancreatic cancer cells *via* stimulating the expression of TGF*β*R1 and TGF*β*R2 (32). TGF*β*R1 and TGF*β*R2 were found to be directly targeted by miR-665. However, Linc00462 sponged away miR-665 and relived inhibitory effects of miR-665 on TGF*β*R1 and TGF*β*R2 (**Figure 1**). linc00462 overexpression significantly increased p-SMAD2 and p-SMAD3 whereas, overexpression of miR-665 significantly decreased p-SMAD2 and p-SMAD3 (32).

PVT1 stimulated TGF*β*/SMAD signaling that sequentially induced epithelial-to-mesenchymal transition (EMT) (32). PVT1 silencing resulted in inactivation of TGF*β*/SMAD signaling *via* reduction of p-SMAD2/3 and TGF*β*1 but there was a notable increase in the levels of SMAD4 (32).

DLEU2 blocked miR-455-mediated targeting of SMAD2 in pancreatic cancer cells **(** **Figure 1**) (33). SMAD2 promoted invasive potential of pancreatic cancer cells. miR-455 overexpression or DLEU2 knockdown significantly suppressed proliferation and invasion of MIA PaCa-2 cells. Similarly, miR-455 inhibition or DLEU2 overexpression significantly induced proliferation and invasion in AsPC-1 cells, whereas SMAD2 inhibition markedly reversed the effects of miR-455 inhibition or DLEU2 overexpression (33).

Collectively, these research reports highlighted oncogenic interplay between lncRNAs and TGFβ/SMAD pathway to promote pancreatic cancer.

### LncRNA-Mediated Regulation of Wnt/*β*-Catenin Signaling

**Figure 2:**
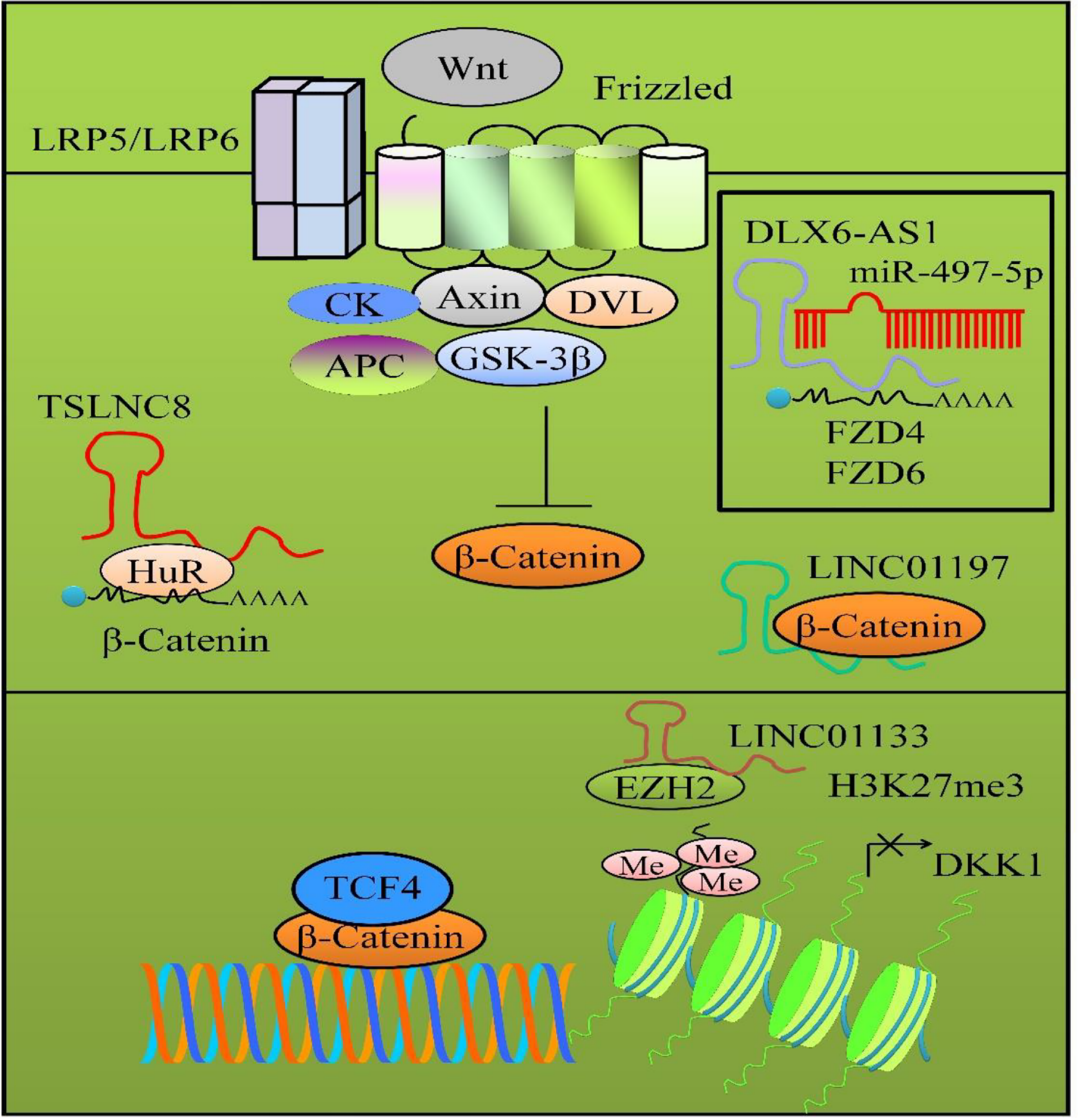
Wnt/*β*-catenin signaling in pancreatic cancer. *β*-catenin moves into the nucleus to transcriptionally modulate wide-ranging target gene networks. LncRNAs regulate different proteins in Wnt/*β*-catenin pathway. LINC01197 physically associated with *β*-catenin and inhibited Wnt/*β*-catenin signaling cascade. DLX6-AS1 interfered with miR-497-5p-mediated targeting of FZD4 and FZD6. TSLNC8 promoted the binding of HuR to *β*-catenin mRNA to stabilize β-catenin. LINC01133 promoted the loading of EZH2 to transcriptionally downregulate DKK1.

FZD4 and FZD6 played central role in activation of Wnt/*β*-catenin pathway. miR-497-5p targeted FZD4 and FZD6 and inhibited Wnt/*β*-catenin transduction cascade (34). DLX6-AS1 interfered with miR-497-5p-mediated targeting of FZD4 and FZD6 (**Figure 2**). DLX6-AS1 knockdown inhibited the metastatic capacity of pancreatic cancer cells by reducing the number of metastatic foci in the liver and lungs. However, DLX6-AS1 overexpression considerably enhanced metastatic foci in the liver and lungs of xenografted mice (34). Overall, these results indicated that DLX6-AS1 acted an oncogenic lncRNA and potentiated Wnt/*β*-catenin signaling.

Linc00261 inhibited the activation of the *β*-catenin/TCF4 pathway and cell metastasis by blocking miR-552-5p-induced targeting of FOXO3 in pancreatic cancer cells (35). *β*-catenin and TCF4 were noted to be reduced in Linc00261-overexpressing pancreatic cancer cells. There was a negative relationship of FOXO3 and *β*-catenin/TCF4 in pancreatic cancer cells. The number of metastatic foci was reduced in the mice injected with the Linc00261-expressing PANC-1 cells (35).

TSLNC8 worked jointly with HuR and promoted the binding of HuR to *β*-catenin mRNA to stabilize *β*-catenin, thus activating WNT/*β*-catenin transduction cascade (**Figure 2**) (36).

HOTAIR inhibition increased the expression of WIF-1 (Wnt inhibitory factor 1) and enhanced radiosensitivity of pancreatic cancer cells (37).

LINC01197 physically associated with *β*-catenin and inhibited Wnt/*β*-catenin signaling cascade in PANC1 and BxPC3 cancer cells (**Figure 2**) (38). LINC01197 disassembled *β*-catenin and TCF4 in BxPC3 and PANC1 cells. LINC01197 overexpression inhibited the binding of *β*-catenin and TCF4 both in BxPC3 and PANC1 cells. LINC01197 overexpression induced significant inhibition of the growth of the tumors derived from BxPC3- and PANC1 cancer cells (38).

DKK1 is a soluble inhibitor of Wnt/*β*-catenin cascade that can bind to LRP5/6 and induce internalization of LRP proteins (39). LINC01133 promoted the loading of EZH2 to transcriptionally downregulate DKK1 in pancreatic cancer cells (**Figure 2**). Importantly, metastatic spread to the liver and lungs was reduced in mice inoculated with LINC01133-silenced pancreatic cancer cells (39).

### Regulation of NOTCH Signaling

SNHG1 has been reported to be significantly upregulated in pancreatic cancer cells (40). NOTCH-induced oncogenic pathway was also noted to be active in pancreatic cancer cells. SNHG1 knockdown exerted inhibitory effects on the activation of the NOTCH-driven signaling pathway and inhibited the expression of NOTCH-1, HES1, vimentin, and N-cadherin (40).

Levels of Jagged-1, HES1, HES5 were noted to be markedly reduced in RP11-567G11.1-depleted PANC-1 and BXPC-3 cells (41). Overall, these findings provided evidence that different long non-coding RNAs effectively potentiated NOTCH-driven pathway in the pancreatic cancer cells.

NOTCH3 is negatively regulated by miR-613 in pancreatic cancer cells (42). HOTAIR sequestered away miR-613 and potentiated NOTCH3 expression. miR-613 overexpression or knockdown of HOTAIR suppressed tumor growth and also reduced the expression of NOTCH3 (42).

### Regulation of JAK/STAT Signaling

STAT1-mediated transduction cascade played critical role in the progression of pancreatic cancer (43). miR-382-3p exerted tumor suppressive effects and directly targeted STAT1. However, PSMB8-AS1 interfered with miR-382-3p-mediated inhibition of STAT1. There was a significant increase in the growth of tumors in experimental mice xenografted with PSMB8-AS1-overexpressing PANC-1 cells. STAT1 and PD-L1 were found to be upregulated in mice xenografted with PSMB8-AS1-overexpressing PANC-1 cells (43).

H19, an oncogenic lncRNA effectively promoted STAT3-mediated signaling in pancreatic cancer cells (44). miR-675 is transcribed from the first exon of H19 and negatively regulates SOCS5 (Suppressor of cytokine signaling 5). SOCS5 is involved in the inhibition of STAT3-driven signaling. It was found that miR-675 negatively modulated SOCS5 and potentiated the expression of STAT3. H19 upregulation reduced gemcitabine chemosensitivity and lowered apoptosis in CAPAN-1 cells. However, gemcitabine chemosensitivity and the apoptosis rate were significantly increased in H19-silenced PANC-1 cells (44).

### Regulation of TRAIL-Mediated Signaling

Cell surface expression of death receptors (DR4 and DR5) is of critical importance to achieve therapeutic effects of TRAIL-based therapeutics.

HOTAIR worked synchronously with EZH2 and transcriptionally downregulated DR5 in pancreatic cancer cells (45). HOTAIR inhibited DR5 transcription by enhancing EZH1-induced histone H3 trimethylation on DR5 gene. HOTAIR knockdown in the TRAIL-resistant PANC-1 cancer cells restored apoptotic cell death (45).

## Mechanistic Interplay Between LncRNAs and Proteins in Pancreatic Cancer

Here we provided a list of lncRNAs reportedly involved in the regulation of myriad of proteins in pancreatic cancer.

## PLACT1

**Figure 3:**
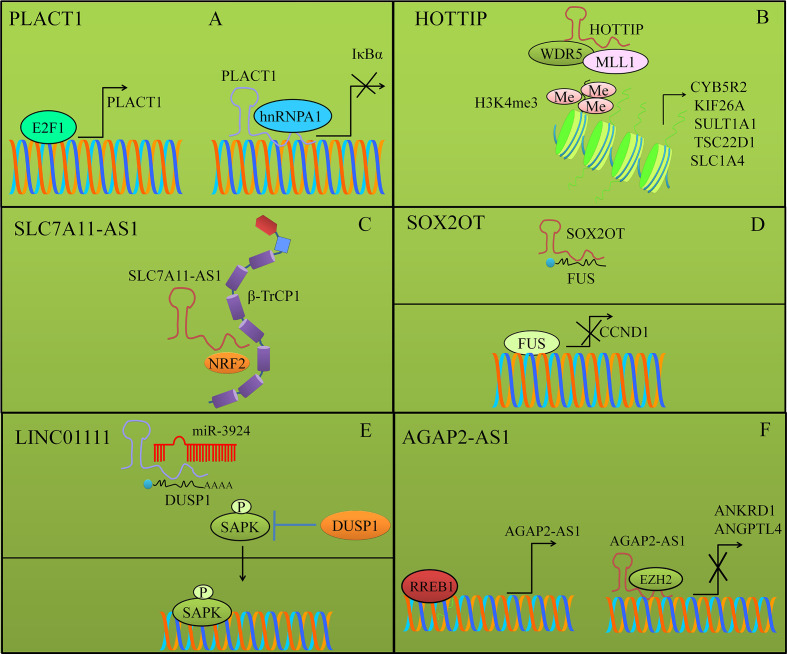
**(A)** PLACT1 mediated inhibition of I*κ*B*α* by epigenetic inactivation. PLACT1 also enhanced loading of hnRNPA1 to the promoter region of I*κ*B*α*. E2F1 induced activation of PLACT1. **(B)** HOTTIP formed a complex with adaptor protein WDR5 and MLL1 (H3K4 methyltransferase) to trans-activate oncogenic proteins by increasing the levels of trimethylated lysine-4 at histone-3 (H3K4) at their promoters. **(C)** SLC7A11-AS1 blocked *β*-TRCP-induced ubiquitination and degradation of NRF2. **(D)** SOX2OT destabilized FUS protein by binding directly to FUS. FUS transcriptionally repressed CCND1. **(E)** DUSP1 (Dual-specificity phosphatase-1) mediated dephosphorylation of SAPK resulted in inhibition of the pathway. LINC01111 sponged away DUSP1 from miR-3924 and promoted expression of DUSP1. DUSP1 dephosphorylated SAPK and prevented its nuclear accumulation. **(F)** RREB1-stimulated expression of AGAP2-AS1. AGAP2-AS1 interacted with EZH2 and repressed expression of ANGPTL4 and ANKRD1.

PLACT1 an oncogenic lncRNA has been described to repress I*κ*B*α* expression mainly through increased loading of hnRNPA1 to promoter region of I*κ*B*α* (46). Additionally, there was an evident increase in the trimethylated levels of lysine 27 of histone-3 that also played role in epigenetic inactivation of I*κ*B*α* (**Figure 3**). E2F1-mediated stimulation of PLACT1 fueled progression of PDAC by sustained activity of NF-*κ*B cascade. Expectedly, use of NF-*κ*B signaling inhibitors caused significant suppression of PLACT1-induced sustained NF-*κ*B activity that consequentially induced regression of tumors in xenografted mice (46).

## HOTTIP

HOTTIP (HOXA transcript at the distal tip) formed a complex with adaptor protein WDR5 and MLL1 (H3K4 methyltransferase) to trans-activate oncogenic proteins CYB5R2, KIF26A, SULT1A1, TSC22D1, and SLC1A4 by increasing the levels of trimethylated lysine-4 at histone-3 (H3K4) at their promoters (**Figure 3**) (47). Collectively, these findings provided concrete evidence of fundamental role of HOTTIP in promotion of PDAC progression through the HOTTIP–WDR5–MLL1 axis.

## SLC7A11-AS1

SLC7A11-AS1 promoted chemoresistance through reduction of intracellular ROS by stabilizing NRF2 (nuclear factor erythroid-2-related factor 2) (48). Proteomic studies revealed that SLC7A11-AS1 co-localized with *β*-TRCP1 in the nucleus. *β*-TrCP, an F-box protein served as substrate-recognition subunit for the SCF*β*–TrCP E3 ubiquitin ligase, which mediated ubiquitylation of a broad range of substrates and post-translationally marked proteins for degradation. A series of experiments revealed that exon 3 of SLC7A11-AS1 interacted with the F-box motif of *β*-TRCP1. The F-box motif of *β*-TRCP1 acted as a critical domain that recruited *β*-TRCP1 to the SCF*β*–TRCP E3 complex. Resultantly, this interaction prevented ubiquitination and degradation of NRF2 in the nucleus. SLC7A11-AS1 overexpression blocked SCFβ–TRCP-induced ubiquitination and degradation of NRF2 and effectively reduced intracellular levels of ROS (**Figure 3**) (48).

## SOX2OT

SOX2OT fueled proliferation capacity of PDAC cells by binding directly to FUS and destabilizing the FUS protein (**Figure 3**). SOX2OT upregulated the proliferation of the BxPC-3 and PANC-1 cells. FUS transcriptionally repressed CCND1 in pancreatic cancer cells (49).

### Interplay Between DANCR and IGF2BP2

N6-methyladenosine (m6A) has been shown to tag wide-ranging messenger RNAs in mammalian cells. Molecular studies had shown that m6A modification machinery consisted of “writers”, “erasers”, and “readers” (50). The YTH domain family (YTHDF) and IGF2BPs belonged to large families of RNA-binding proteins (RBPs) and served as readers. IGF2BPs served as a specialized family of m6A readers that targeted various mRNA transcripts. IGF2BPs stabilized and stored mRNAs marked by m6A during stress and normal situations (50). IGF2BP2 promoted pancreatic cancer cell proliferation. DANCR expression was upregulated in IGF2BP2-overexpressing cancer cells. Moreover, knockdown of IGF2BP2 suppressed DANCR expression. DANCR knockdown suppressed cell proliferation and colony formation. IGF2BP2 interacted with DANCR and stabilized it effectively. RNA methylation (m6A) occurred at 664^th^ nucleotide of DANCR. Methylated DANCR was recognized by IGF2BP2 and resultantly, IGF2BP2 served as a reader for the methylated version of DANCR and increased its stability (50).

## LINC01111

LINC01111 acted as a tumor suppressor lncRNA in pancreatic cancer. LINC01111 knockdown enhanced cell proliferation, invasion, and migration *in vitro* (51). Tumor growth was found to be significantly enhanced in mice xenografted with LINC01111-silenced pancreatic cancer cells. Higher expression levels of LINC01111 relieved repressive effects of miR-3924 on DUSP1 and effectively blocked SAPK phosphorylation and thus inactivated SAPK/JNK signaling pathway in pancreatic cancer cells. Shown in **Figure 3**. DUSP1 (Dual-specificity phosphatase-1) is reportedly involved in dephosphorylation of different proteins. Therefore, DUSP1-mediated dephosphorylation of SAPK resulted in inhibition of the pathway (**Figure 3**). Functional studies had shown that phosphorylation activated function of SAPK/JNKs. SAPK/JNKs translocated from the cytoplasm to the nucleus where they phosphorylated series of genes including c-Jun, ATF2, *etc.* and dramatically enhanced their transcriptional activities (51).

## AGAP2-AS1

RREB1-binding sites have been identified in the promoter region of AGAP2-AS1 and consequently could binding of RREB1 to the promoter region of AGAP2-AS1 stimulated its expression (52). AGAP2-AS1 worked synchronously with EZH2 (enhancer of zeste homolog-2) and epigenetically inhibited ANGPTL4 and ANKRD1 and fueled proliferation and metastasis of pancreatic cancer cells (52). Shown in **Figure 3**.

## XLOC_006390

Overexpression of XLOC_006390 promoted the protein stability of c-Myc by blocking its ubiquitination. c-Myc transcriptionally upregulated glutamate dehydrogenase-1 in pancreatic cancer cells (53, 54). Collectively, XLOC_006390 promoted pancreatic carcinogenesis and glutamate metabolism by stabilization of c-Myc (53, 54).

## LINC01638

LINC01638 overexpression promoted, while LINC01638 silencing inhibited migratory and invasive potential of PDAC cell line (55, 56). LINC01638 overexpression increased TGF*β*1, while silencing of LINC01638 markedly reduced TGF*β*1 expression in pancreatic ductal adenocarcinoma cell line (55, 56).

## MACC1-AS1

MACC1-AS1 acted an oncogenic lncRNA and potentiated the expression of SMAD2 by sequestering it away from miR-145 (57, 58). In turn, SMAD2 stimulated the expression of MACC1-AS1 by directly binding to the promoter. Overall, these results clearly suggested that MACC1-AS1 promoted cancer by potentiating SMAD-driven signaling.

**Figure 4:**
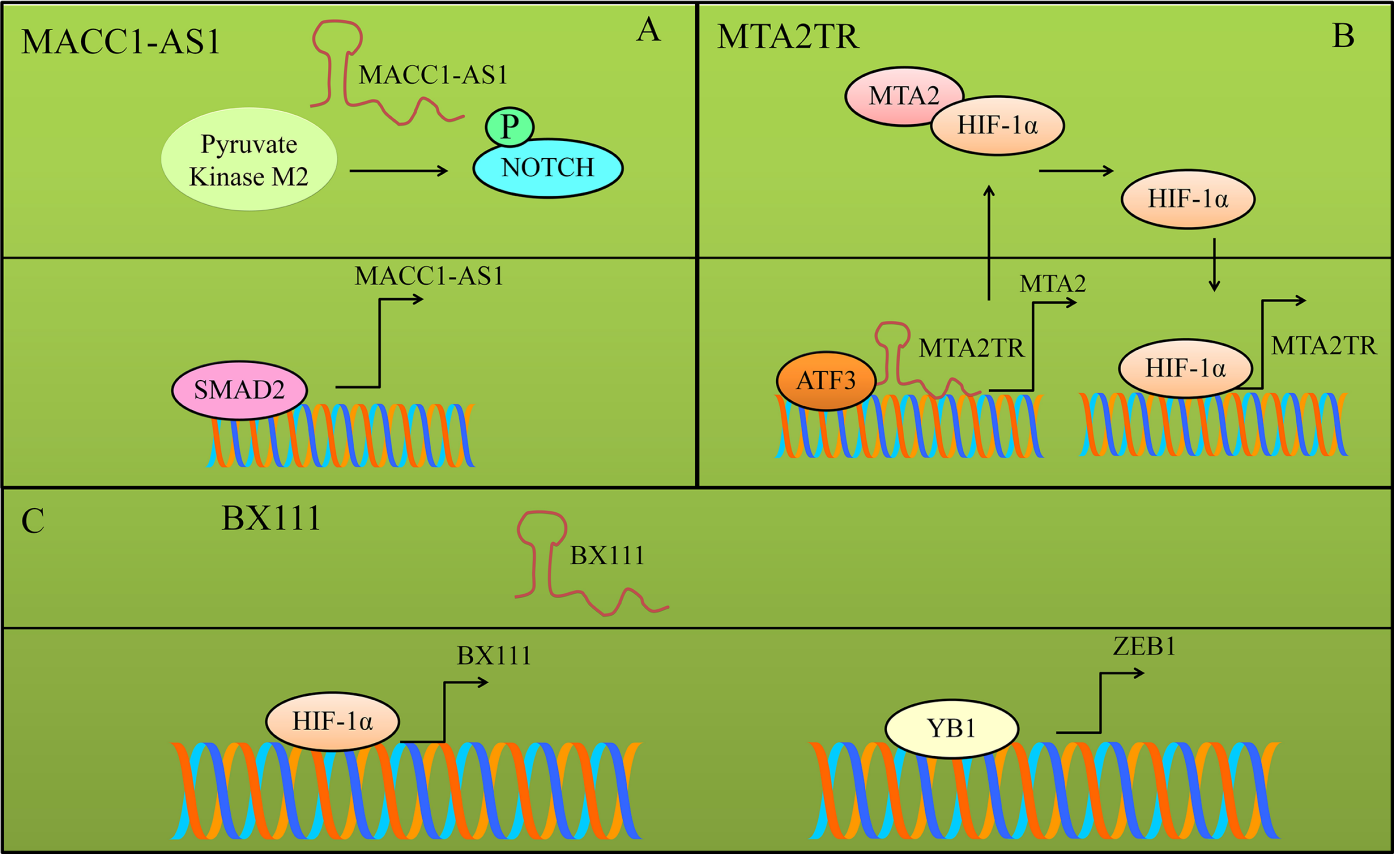
Different proteins transcriptionally upregulated the expression of various lncRNAs. **(A)** SMAD2 transcriptionally upregulated MACC1-AS1. MACC1-AS1 stabilized and promoted pyruvate kinase M2-driven phosphorylation of NOTCH1. Oncogenic NOTCH1 pathway was activated by MACC1-AS1 through pyruvate kinase M2. **(B)** MTA2TR and ATF3 assembled in the nucleus and stimulated the expression of MTA2. MTA2 stabilized HIF protein and consequently HIF transcriptionally activated MTA2TR. **(C)** BX111 was transcriptionally stimulated by HIF-1*α*. Furthermore, BX111 facilitated the binding of YB1 to promoter region of ZEB1.

MACC1-AS1 knockdown inhibited the proliferation as well as metastasizing capacity of pancreatic cancer cells. MACC1-AS1 overexpressing pancreatic cancer cells demonstrated significantly increased mobility of cancer cells (53, 54). MACC1-AS1 stabilized protein levels of pyruvate kinase M2. NOTCH1 phosphorylation was increased in MACC1-AS1-overexpressing pancreatic cancer cells. However, phosphorylation was blocked in pyruvate kinase M2-knockdown cancer cells (53, 54). Overall, these findings suggested that MACC1-AS1 potentiated pyruvate kinase M2-driven phosphorylation of NOTCH1 (**Figure 4**). Oncogenic NOTCH1 pathway was activated by MACC1-AS1 through pyruvate kinase M2 in the pancreatic cancer cells.

## MTA2TR

Metastasis associated protein 2 (MTA2) transcriptional regulator lncRNA (MTA2TR) worked synchronously with ATF3 (activating transcription factor-3) and transcriptionally upregulated the expression of MTA2 (55, 56). MTA2 stabilized HIF-1*α* protein *via* deacetylation and promoted HIF-1*α*-induced transcriptional upregulation of MTA2TR (56). Shown in **Figure 4**. Overall, these results highlighted intricate role of MTA2TR in stabilization of HIF-1*α via* MTA2 in pancreatic cancer cells.

## PCTST

lnc-PCTST, a tumor suppressor lncRNA increased E-cadherin and simultaneously reduced vimentin levels (57, 58). Additionally, TACC-3 knockdown also induced an increase in the levels of E-cadherin. Functional studies revealed that lnc-PCTST was closely associated with its genomic neighboring gene TACC-3 and considerably reduced its promoter activity (57, 58).

## HOXA-AS2

Detailed mechanistic insights revealed that HOXA-AS2 interacted directly with EZH2 (enhancer of zeste homolog-2) and lysine specific demethylase 1 (LSD1) and synchronously promoted growth ability of pancreatic cancer cells (59).

## KCNK15-AS1

KCNK15-AS1 markedly reduced migratory and invasive potential of BxPC-3 and MIA PaCa-2 cells (60). KCNK15-AS1 m6A enrichment was noted to be significantly higher in BxPC-3 and MIA PaCa-2 cells. ALKBH5, a versatile RNA m6A demethylase efficiently demethylated KCNK15-AS1 in pancreatic cancer cells (60). Overall, these results indicated that ALKBH5-driven demethylation of KCNK15-AS1 dramatically reduced migratory and invasive potential of pancreatic cancer cells.

## BX111

BX111 was transcriptionally stimulated by HIF-1*α* (hypoxia-inducible factor) in response to hypoxia (61). Furthermore, BX111 participated in hypoxia-driven EMT of pancreatic cancer cells by promoting the binding of Y-box protein (YB1) to promoter region of ZEB1 (**Figure 4**) (61).

## HOTAIR

HOTAIR is an oncogenic lncRNA and increased expression of HOTAIR is indicative of a poor prognosis in cancer patients (62). Although its exact role is not fully understood, several studies have revealed some of its functions in pancreatic cancer. A recent study (63) showed that HOTAIR serves as up-stream regulator of HK2, an enzyme that catalyzes the first step of glycolysis (64) and thus boosting cancer cell proliferation in pancreatic cancer cells. Its overexpression also increases both ATP and lactate production as well as glucose uptake. Additionally, increased HOTAIR levels downregulated death receptor 5 (DR5) and prevented TRAIL-mediated apoptosis. EZH2 effectively catalyzed histone H3 lysine 27 trimethylation (H3K27me3), an essential epigenetic modification on histone that controlled structure of the chromatin and epigenetically silenced target genes. HOTAIR knockdown markedly decreased H3K27me3 loading to the DR5 promoter, while HOTAIR overexpression greatly enhanced H3K27me3 loading to the DR5 promoter. HOTAIR worked synchronously with EZH2 and marked promoter of DR5 with H3K27me3 to epigenetically silence DR5 (45). Moreover, polymorphisms such as rs4759314 and rs200349340 increase HOTAIR expression and promote pancreatic cancer susceptibility (65, 66).

## MALAT1

MALAT1 and EZH2 epigenetically inactivated E-cadherin and potently enhanced invasion and migration of pancreatic cancer cells (67). Importantly, histone methylation and DNA methylation are highly dynamic mechanisms centrally involved in the reprogramming of gene networks in wide variety of cellular processes. Different chemicals are currently being tested to inhibit polycomb repressive complexes to re-program gene networks (68, 69). Furthermore, knockdown of MALAT1 inhibited proliferation, migration, invasion as well as the expression of genes involved in EMT. Knockdown of MALAT1 also results in downregulation of Snail and Slug, two transcription factors that are related to EMT (70).

### Competing Endogenous RNA Activity of LncRNAs

Wealth of information has portrayed competing endogenous RNA (ceRNA) activity as a large-scale regulatory network across the transcriptome which has greatly expanded the functional genetic information in the human genome and played role in cancer onset and progression.

Linc00976 promoted proliferation, migration, and invasion by sequestering OTUD7B away from miR-137 in pancreatic cancer cells (71). OTUD7B, a deubiquitination enzyme efficiently deubiquitinated EGFR and activated downstream pathway. EGFR was considerably more stable in OTUD7B-overexpressing pancreatic cancer cells (71).

LOXL1-AS1 promoted pancreatic cancer by promoting the expression of Semaphorin 7A (SEMA7A) and sequestering it away from miR-28-5p (72).

Cancer susceptibility candidate 2 (CASC2) exerted tumor-suppressive effects through regulation of miR-24/MUC6 axis in pancreatic cancer cells (73). miR-24 knockdown or CASC2 overexpression suppressed pancreatic cancer cell proliferation, migration, invasion and promoted apoptosis. Mechanistically, CASC2 sponged miR-24 and relieved the repressive effects of miR-24 on MUC6 to suppress pancreatic cancer growth and progression (73).

LINC00657, an oncogenic lncRNA promoted the expression of PAK4 (p21 activated kinase-4) by protecting it from miR-433 (74). Tumor growth was significantly reduced in mice xenografted with LINC00657-silenced PDAC cells (74).

Long intergenic non-coding RNA for kinase activation (LINK-A) acted as an oncogenic lncRNA and promoted migratory and invasive potential of BxPC-3 *via* stimulation of ROCK1 (Rho associated coiled-coil containing protein kinase-1) (75).

### Role of Long Non-Coding RNAs in Regulation of Drug Resistance in Pancreatic Cancer

HCP5, an lncRNA, stimulated the expression of hepatoma-derived growth factor (HDGF) by protecting it from targeting by miR-214-3p. HCP and HDGF promoted gemcitabine resistance in pancreatic cancer cells (76).

SBF2-AS1 knockdown inhibited proliferation, EMT and induction of apoptotic cell death in gemcitabine-resistant pancreatic cancer cells. SBF2-AS1 potentiated the expression of TWF1 by sponging away miR-142-3p (77).

The recent resurgence of public interest in herbal remedies, it was report that ginsenoside Rg3 effectively induced apoptosis in gemcitabine-resistant pancreatic cancer cells. Essentially, levels of CASC2 and PTEN were found to be considerably elevated in ginsenoside-treated pancreatic cancer cells (78).

### Diagnostic Potential of LncRNAs

Because of better detection and high specificity in the liquid biopsy and tissue, there is increasing interest in exploring the potential of lncRNAs in cancer patients (79, 80). Profiling of the lncRNAs derived from extracellular vesicles has helped in the identification of a diagnostic signature for the detection of pancreatic ductal adenocarcinoma (81).

## Concluding Remarks

Non-coding RNA biology has exploded in the recent era, and we have witnessed overwhelmingly increasing list of miRNAs and lncRNAs which regulated cancer onset and progression. Additionally, the concept of ceRNA has leveraged our understanding of the layered regulatory network of lncRNAs to another level. Cellular and molecular biologists are now focusing on identification of specialized lncRNAs which play crucial role in pancreatic cancer. Identification of most relevant lncRNAs will enable the development of mimics and antisense oligonucleotides for efficient treatment of pancreatic cancer.

## Author Contributions

SN, CM, and HK gathered and wrote the initial draft. AF, RB, MG, and WC input and edited the draft. AF designed the diagrams. AF, RB, MG, and WC checked and polished the diagrams. All authors contributed to the article and approved the submitted version.

## Conflict of Interest

The authors declare that the research was conducted in the absence of any commercial or financial relationships that could be construed as a potential conflict of interest.
